# Hepatic transcriptome analysis reveals altered lipid metabolism and consequent health indices in chicken supplemented with dietary *Bifidobacterium bifidum* and mannan-oligosaccharides

**DOI:** 10.1038/s41598-021-97467-1

**Published:** 2021-09-09

**Authors:** Kapil Dev, Jubeda Begum, Avishek Biswas, Nasir Akbar Mir, Jitendra Singh, Ravi Prakash, Joyshikh Sonowal, Krishna Bharali, Simmi Tomar, Rajiv Kant, Neeraj Ahlawat

**Affiliations:** 1 ICAR-Central Avian Research Institute, Izatnagar, Bareilly, 243122 India; 2 College of Veterinary & Animal Sciences, Govind Ballabh Pant University of Agriculture & Technology, Pantnagar, 263145 India; 3 CSIR-Central Drug Research Institute, Lucknow, 226031 India; 4 ICAR-Indian Veterinary Research Institute, Izatnagar, Bareilly, 243122 India; 5 Sam Higginbottom University of Agriculture, Technology and Sciences, Prayagraj (Allahabad), 211007 India

**Keywords:** Biochemistry, Biotechnology, Microbiology, Molecular biology

## Abstract

This study investigated the role of dietary prebiotic mannan-oligosaccharides (MOS), and probiotic *Bifidobacterium bifidum* (BFD) in lipid metabolism, deposition, and consequent health indices in broiler chicken. The supplementation of 0.2% MOS along with either 10^6^ or 10^7^ CFU BFD/g feed resulted in downregulation of Acetyl-CoA carboxylase, fatty acid synthase, sterolregulatory element binding protein-1, and apolipoprotein B100; and up-regulation of peroxisome proliferator activated receptor-α AMP-activated protein kinase α-1, and stearoyl CoA (∆9) desaturase-1 hepatic expression in broiler chicken. The birds supplemented with 0.2% MOS along with either 10^6^ or 10^7^ CFU BFD/g feed depicted lower body fat percentage, palmitic acid, stearic acid, and saturated fatty acid contents, whereas, higher palmitoleic acid, oleic acid, and MUFA contents were observed. The ∆9-desaturase indices of chicken meat have shown higher values; and elongase index (only thigh) and thioesterase index have shown lower values in birds supplemented with 0.2% MOS along with either 10^6^ or 10^7^ CFU BFD/g feed. The meat health indices such as Polyunsaturated fatty acids (PUFA)/Saturated fatty acids (SFA) ratio, Mono-saturated fatty acids (MUFA)/SFA ratio, unsaturated fatty acids (UFA)/SFA ratio, hypocholesterolemic/hypercholesterolemic fatty acid ratio, saturation index, atherogenic index, thrombogenic index, and hypercholesterolemic fatty acid content were positively improved in birds supplemented with 0.2% MOS along with either 10^6^ or 10^7^ CFU BFD/g feed. Similarly, the birds supplemented with 0.2% MOS along with either 10^6^ or 10^7^ CFU BFD/g feed have shown lower serum triglyceride and total cholesterol levels along with higher high density levels and improved serum health indices cardiac risk ratio, atherogenic coefficient, and, atherogenic index of plasma.

## Introduction

The major organ of intermediary metabolism of lipids in broiler chicken is liver^1,2^. A balance between lipogenesis and lipolysis determines the rate of lipid deposition in broiler chicken^3,4^. A coordinated action of a series of genes involved in lipogenesis, lipolysis, transport, and deposition of lipids, such as acetyl-CoA carboxylase (ACC), fatty acid synthase (FAS), sterol regulatory element binding protein-1c (SREBP-1c), malic enzyme (ME), stearoyl CoA (Δ9) desaturase 1 (SCD-1), peroxisome proliferator activated receptor-α (PPAR-α), AMP-activated protein kinase (AMPK), and apolipoprotein B100 (apoB100) result in a net fat accretion in the body of broiler chicken^3,5,6^. Furthermore, the development of adipose in broiler chicken is determined by the serum triglyceride levels and the major substrates of lipid metabolism are triglycerides along with cholesterol fractions^6^. All these genes involved in lipogenesis, lipolysis, and transport of lipids are responsive to nutritional interventions directly or indirectly which results in alteration of lipid metabolism and the consequent lipid deposition in chicken^3,6,7^.

One of the prominent nutritional interventions in broiler chicken is the supplementation of dietary prebiotics and probiotics for desired growth, immunity, and meat quality of birds^8,9^; and recent studies have focussed on the importance of prebiotics and probiotics in the regulation of lipid metabolism in broiler chicken^3,10–12^. The supplementation of prebiotics in broiler chicken foster the population of lactic acid producing bacteria of *Bifidobacterium* and *Lactobacillus* genera^4,13^. The probiotic strains have the potential to regulate lipid metabolism in chicken by altering the expression of genes involved in lipogenesis, lipolysis, and transport of lipids which elicit hypolipidemic effects and enhance the health value of chicken meat^10,12–16^.

Furthermore, modern broiler chicken strains are hyperphagic in nature and tend to deposit more fat in body^3^. But, the excess fat deposition in chicken is a double facet problem—firstly, it is an economic burden on poultry producers which is discarded while processingand causes waste management problems, secondly, it is highly associated with cardiovascular diseases in humans^17,18^. However, the supplementation of synbiotics—prebiotic and probiotic combinations, have shown hypolipidemic and hypocholesterolemic effects^3,13,19–21^. Therefore, this study investigated the role of dietary prebiotic mannan-oligosaccharides (MOS), and probiotic *Bifidobacterium bifidum* (BFD) in lipid metabolism, deposition, and consequent health indices in broiler chicken.

## Results

### Hepatic gene expression

**Figure 1:**
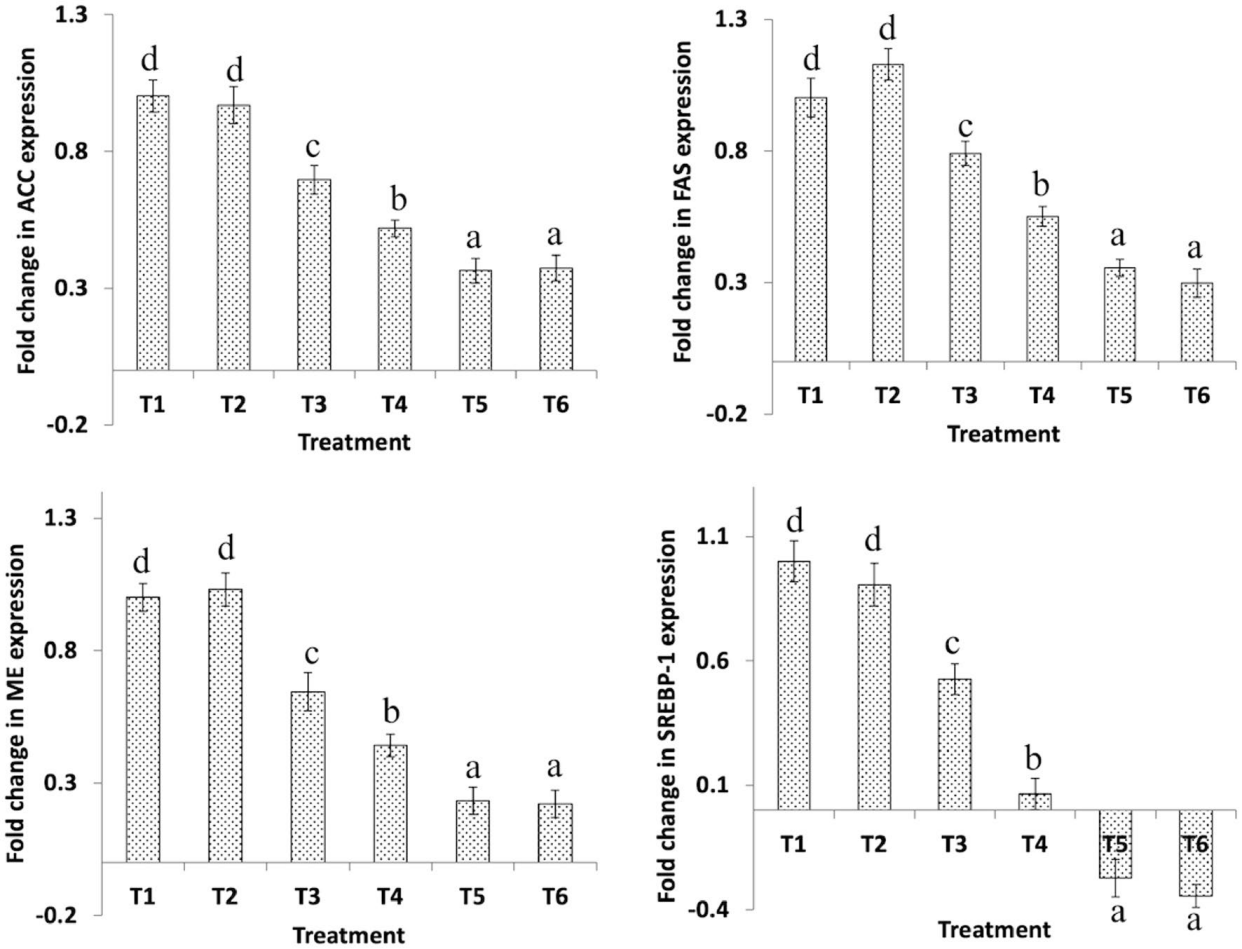
Effect of dietary *B. Bifidum* (BFD) and mannan-oligosaccharides (MOS) on hepatic ACC, FAS, ME, and SREBP-1 gene expression in 21 days old broiler chicken. T_1_ (no BFD/MOS/BMD), T_2_ (20 mg BMD/kg diet), T_3_ (0.1% MOS + 10^6^ cfu BFD/g feed), T_4_ (0.1% MOS + 10^7^ cfu BFD/g feed), T_5_ (0.2% MOS + 10^6^ cfu BFD/g feed), T_6_ (0.2% MOS + 10^7^ cfu BFD/g feed). Results are presented as means ± SEM with six birds per treatment. Means values with different superscripts letters differ significantly (P < 0.05).

**Figure 2:**
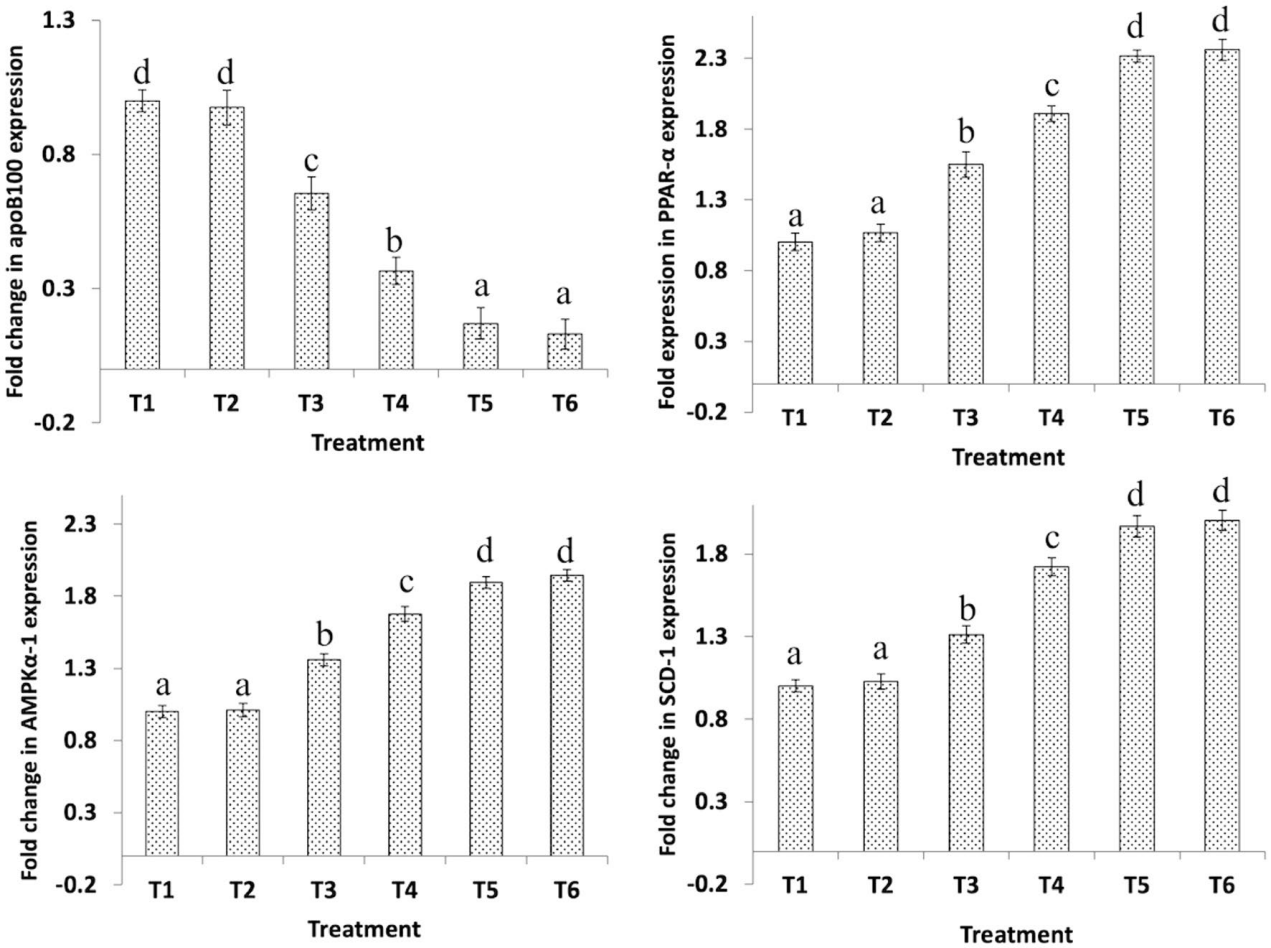
Effect of dietary *B. Bifidum* (BFD) and mannan-oligosaccharides (MOS) on hepatic apoB100, PPAR-α, AMPKα-1, and SCD-1 gene expression in 21 days old broiler chicken. T_1_ (no BFD/MOS/BMD), T_2_ (20 mg BMD/kg diet), T_3_ (0.1% MOS + 10^6^ cfu BFD/g feed), T_4_ (0.1% MOS + 10^7^ cfu BFD/g feed), T_5_ (0.2% MOS + 10^6^ cfu BFD/g feed), T_6_ (0.2% MOS + 10^7^ cfu BFD/g feed). Results are presented as means ± SEM with six birds per treatment. Means values with different superscripts letters differ significantly (P < 0.05).

**Figure 3:**
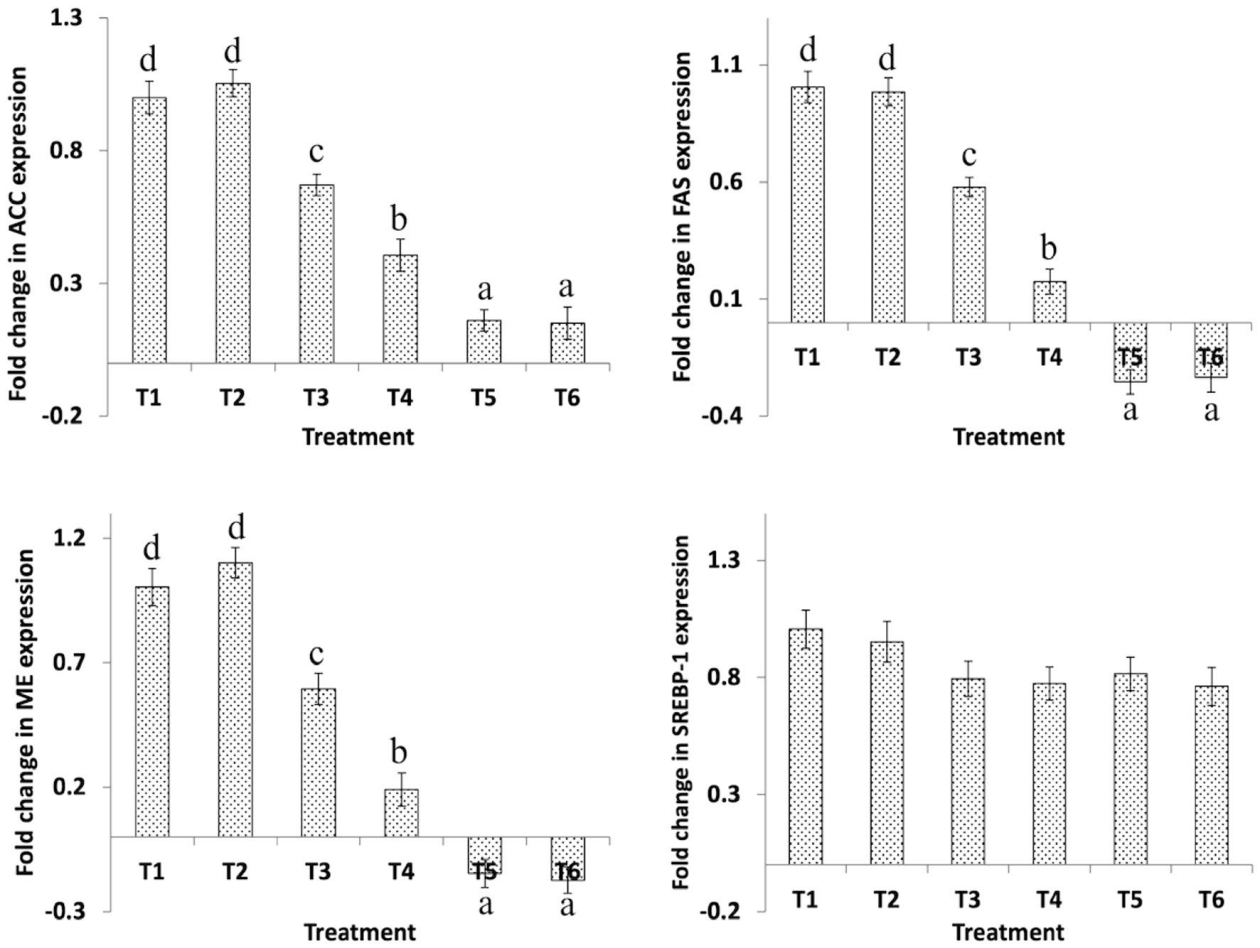
Effect of dietary *B. Bifidum* (BFD) and mannan-oligosaccharides (MOS) on hepatic ACC, FAS, ME, and SREBP-1 gene expression in 42 days old broiler chicken. T_1_ (no BFD/MOS/BMD), T_2_ (20 mg BMD/kg diet), T_3_ (0.1% MOS + 10^6^ cfu BFD/g feed), T_4_ (0.1% MOS + 10^7^ cfu BFD/g feed), T_5_ (0.2% MOS + 10^6^ cfu BFD/g feed), T_6_ (0.2% MOS + 10^7^ cfu BFD/g feed). Results are presented as means ± SEM with six birds per treatment. Means values with different superscripts letters differ significantly (P < 0.05).

**Figure 4:**
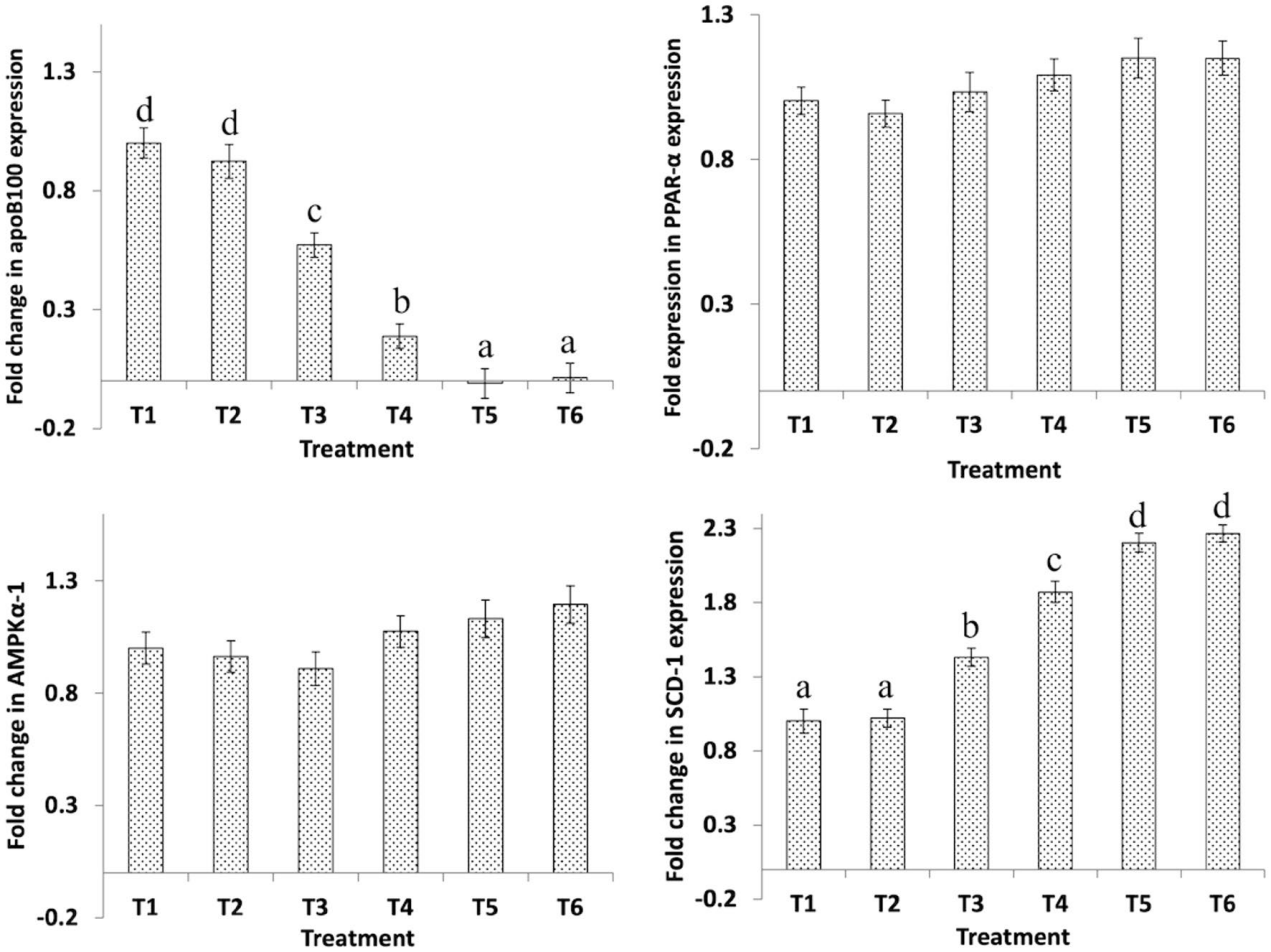
Effect of dietary *B. Bifidum* (BFD) and mannan-oligosaccharides (MOS) on hepatic apoB100, PPAR-α, AMPKα-1, and SCD-1 gene expression in 42 days old broiler chicken. T_1_ (no BFD/MOS/BMD), T_2_ (20 mg BMD/kg diet), T_3_ (0.1% MOS + 10^6^ cfu BFD/g feed), T_4_ (0.1% MOS + 10^7^ cfu BFD/g feed), T_5_ (0.2% MOS + 10^6^ cfu BFD/g feed), T_6_ (0.2% MOS + 10^7^ cfu BFD/g feed). Results are presented as means ± SEM with six birds per treatment. Means values with different superscripts letters differ significantly (P < 0.05).

The relative expression pattern of genes involved in lipid metabolism of broiler chicken under the influence of BFD and MOS are given in Figs. 1, 2, 3, and 4. The expression pattern of ACC, FAS, ME, SERBP-1 (21 days only), and apoB100 depicted a progressive (P < 0.05) decrease from T1 (control) to T6. There were no significant differences between T1 and T2; and T5 and T6. Also, the expression pattern of SERBP-1 in broiler chicken at 42 days of age did not show any significant dietary effects. However, the expression pattern of PPAR-α, AMPKα-1 (21 days only), and SCD-1 revealed an increasing trend from T1 (control) to T6. Again, no significant differences were observed between T1 and T2; and T5 and T6. The expression of AMPKα-1 at 42 days of was not affected by dietary treatments.

### Fat percentage and fatty acid profile of chicken meat

The results given in Tables 1 and 2 reveal significant effects of BFD and MOS supplementation on fat percentage and some constituent fatty acids of broiler chicken breast and thigh at 42 days of age, respectively. However, it is noteworthy to point out that CARIBRO Vishal tends to deposit more fat compared to other commercial chicken. From T1 (control) to T6 a significant decreasing trend was observed in percentages of fat (P < 0.01), palmitic acid (P < 0.01), stearic acid (P < 0.05), and SFA (P < 0.05). However, increasing trend was observed in percentage of palmitoleic acid (P < 0.01), oleic acid (P < 0.05), and MUFA (P < 0.01) in chicken breast and thigh meat from treatment T1 to T6. But, no significant differences were observed between T1 and T2; and T5 and T6. The other constituent fatty acids and PUFA were not affected by dietary treatments. Similar pattern was observed at 21 days of age (data not shown).

**Table 1:** Effect of dietary supplementation of *Bifidobacterium bifidum* (BFD) and Mannan-oligosaccharides (MOS) on fatty acid profile of broiler chicken breast muscle (n = 6 × 2).

Fatty acid (%)	T1*	T2*	T3*	T4*	T5*	T6*	SEM	P value
Total fat	4.23^d^	4.14^d^	3.78^c^	3.53^b^	2.94^a^	2.92^a^	0.071	P < 0.01
C14:0	0.61	0.63	0.60	0.61	0.62	0.63	0.023	P > 0.05
C16:0	25.6^d^	25.3^d^	23.2^c^	21.2^b^	19.5^a^	19.2^a^	0.29	P < 0.01
C16:1	1.33^a^	1.51^a^	2.40^b^	3.29^c^	3.98^d^	4.07^d^	0.132	P < 0.01
C18:0	15.6^d^	15.1^d^	13.9^c^	12.2^b^	10.6^a^	10.8^a^	0.27	P < 0.05
C18:1 ω-9	32.4^a^	33.0^a^	34.7^b^	36.9^c^	39.9^d^	39.8^d^	0.39	P < 0.05
C18:2 ω-6	16.2	16.2	16.7	17.0	16.5	16.6	0.413	P > 0.05
C18:3 ω-3	1.01	1.05	1.12	1.18	1.20	1.17	0.112	P > 0.05
C18:3 ω-6	0.57	0.54	0.56	0.58	0.57	0.69	0.142	P > 0.05
C20:0	0.87	0.79	0.79	0.81	0.82	0.84	0.059	P > 0.05
C20:1 ω-9	1.85	1.91	1.84	1.84	1.89	1.93	0.091	P > 0.05
C20:2 ω-6	0.28	0.29	0.31	0.29	0.30	0.32	0.027	P > 0.05
C20:3 ω-3	0.68	0.68	0.72	0.74	0.72	0.74	0.054	P > 0.05
C20:4 ω-6	0.41	0.45	0.43	0.44	0.46	0.47	0.041	P > 0.05
C20:5 ω-3	0.45	0.40	0.44	0.45	0.46	0.47	0.039	P > 0.05
C22:0	0.40	0.45	0.42	0.41	0.45	0.43	0.032	P > 0.05
C22:5 ω-3	0.59	0.64	0.65	0.69	0.67	0.66	0.057	P > 0.05
C22:6 ω -3	1.02	1.09	1.10	1.12	1.20	1.20	0.079	P > 0.05
C24:0	0.12	0.11	0.16	0.14	0.09	0.11	0.014	P > 0.05
SFA	43.2^d^	42.3^d^	39.1^c^	35.5^b^	32.1^a^	32.0^a^	0.64	P < 0.01
MUFA	35.5^a^	36.4^a^	38.9^b^	42.0^c^	45.7^d^	45.8^d^	0.92	P < 0.01
PUFA	19.1	19.2	19.8	20.2	19.9	19.9	0.56	P > 0.05
ω-3 PUFA	2.48	2.55	2.66	2.75	2.85	2.84	0.241	P > 0.05
ω-6 PUFA	16.7	16.6	17.2	17.4	17.0	17.1	0.47	P > 0.05

*T1 (no BFD/MOS/BMD), T2 (20 mg BMD/kg diet), T3 (0.1% MOS + 10^6^ cfu BFD/g feed), T4 (0.1% MOS + 10^7^ cfu BFD/g feed), T5 (0.2% MOS + 10^6^ cfu BFD/g feed), T6 (0.2% MOS + 10^7^ cfu BFD/g feed).

*BMD* Bacitracin Methylene Disalicylate, *SFA* Saturated fatty acids, *MUFA* Monounsaturated fatty acids, *PUFA* Polyunsaturated fatty acids, *SEM* Standard error of mean.

Mean values bearing the same superscripts in a row do not differ significantly.

**Table 2:** Effect of dietary supplementation of *Bifidobacterium bifidum* (BFD) and Mannan-oligosaccharides (MOS) on fatty acid profile of broiler chicken thigh muscle (n = 6 × 2).

Fatty acid (%)	T1*	T2*	T3*	T4*	T5*	T6*	SEM	P value
Total fat	7.02^d^	7.10^d^	6.48^c^	6.09^b^	5.52^a^	5.47^a^	0.061	P < 0.01
C14:0	0.79	0.81	0.78	0.79	0.82	0.80	0.037	P > 0.05
C16:0	24.2^c^	24.0^c^	22.4^b^	21.3^b^	20.4^a^	20.2^a^	0.27	P < 0.01
C16:1	3.03^a^	3.17^a^	4.15^b^	4.82^c^	5.38^d^	5.46^d^	0.147	P < 0.01
C18:0	14.9^d^	14.8^d^	12.2^c^	10.7^b^	9.5^a^	9.8^a^	0.26	P < 0.01
C18:1 ω-9	35.5^a^	35.2^a^	37.9^b^	39.7^c^	41.4^d^	41.2^d^	0.51	P < 0.05
C18:2 ω-6	13.5	13.7	14.2	14.2	14.0	14.0	0.417	P > 0.05
C18:3 ω-3	1.44	1.46	1.50	1.52	1.49	1.52	0.098	P > 0.05
C18:3 ω-6	0.57	0.54	0.58	0.57	0.59	0.58	0.098	P > 0.05
C20:0	0.20	0.20	0.21	0.21	0.20	0.19	0.019	P > 0.05
C20:1 ω-9	1.37	1.36	1.40	1.40	1.41	1.42	0.127	P > 0.05
C20:2 ω-6	0.28	0.29	0.28	0.30	0.31	0.31	0.049	P > 0.05
C20:3 ω-3	0.68	0.68	0.70	0.76	0.77	0.75	0.114	P > 0.05
C20:4 ω-6	0.19	0.19	0.21	0.21	0.22	0.23	0.024	P > 0.05
C20:5 ω-3	0.61	0.64	0.65	0.67	0.69	0.68	0.059	P > 0.05
C22:0	0.62	0.61	0.58	0.57	0.62	0.60	0.041	P > 0.05
C22:5 ω-3	0.69	0.74	0.70	0.71	0.76	0.76	0.096	P > 0.05
C22:6 ω -3	1.23	1.27	1.23	1.22	1.21	1.21	0.059	P > 0.05
C24:0	0.25	0.26	0.23	0.22	0.22	0.22	0.027	P > 0.05
SFA	41.0^d^	40.7^d^	36.4^c^	33.8^b^	31.8^a^	31.9^a^	0.93	P < 0.01
MUFA	39.9^a^	39.8^a^	43.5^b^	45.9^c^	48.2^d^	48.1^d^	0.97	P < 0.01
PUFA	16.9	17.3	17.8	17.8	17.6	17.6	0.64	P > 0.05
ω-3 PUFA	3.28	3.38	3.39	3.41	3.39	3.41	0.124	P > 0.05
ω-6 PUFA	13.7	13.9	14.4	14.4	14.2	14.2	0.49	P > 0.05

*T1 (no BFD/MOS/BMD), T2 (20 mg BMD/kg diet), T3 (0.1% MOS + 10^6^ cfu BFD/g feed), T4 (0.1% MOS + 10^7^ cfu BFD/g feed), T5 (0.2% MOS + 10^6^ cfuB FD/g feed), T6 (0.2% MOS + 10^7^ cfu BFD/g feed).

*BMD* Bacitracin Methylene Disalicylate, *SFA* Saturated fatty acids, *MUFA* Monounsaturated fatty acids, *PUFA* Polyunsaturated fatty acids, *SEM* Standard error of mean.

Mean values bearing the same superscripts in a row do not differ significantly.

### Lipid metabolism and health related indices

The product: precursor ratios were used to assess the activities of various enzymes involved in lipid metabolism. The enzyme activity indices at 42 days of age have revealed a progressive increase (P < 0.01) in ∆9-DI (18), ∆9-DI (16), and total DI; and a progressive decrease (P < 0.05) was observed in thioesterase index and elongase index (thigh only) from treatment T1 (control) to T6 in chicken meat (Table 3). The ∆5 + ∆6 Desaturase index and elongase index (breast only) did not reveal any significant treatment effect and no significant differences were observed between T1 and T2; and T5 and T6.

**Table 3:** Effect of dietary supplementation of *Bifidobacterium bifidum* (BFD) and Mannan-oligosaccharides (MOS) on lipid metabolism indices of broiler chicken (n = 6 × 2).

Indices	T1*	T2*	T3*	T4*	T5*	T6*	SEM	P value
**Breast**								
∆9-DI (18)	67.5^a^	68.6^a^	71.4^b^	75.1^c^	79.0^d^	78.7^d^	0.59	P < 0.01
∆9-DI (16)	4.92^a^	5.63^a^	9.4^b^	13.4^c^	16.9^d^	17.5^d^	0.526	P < 0.01
Total DI^1^	45.0^a^	46.1^a^	50.0^b^	54.6^c^	59.3^d^	59.4^d^	0.71	P < 0.01
Elongase index	0.61	0.60	0.60	0.58	0.54	0.56	0.053	P > 0.05
Thioesterase index	41.9^d^	39.8^ cd^	38.3^c^	34.5^b^	31.4^a^	30.4^a^	1.45	P < 0.05
∆5 + ∆6 desaturase index	13.7	14.3	14.1	14.2	14.8	14.9	0.96	P > 0.05
**Thigh**								
∆9-DI (18)	70.4^a^	70.4^a^	75.1^b^	78.3^c^	81.3^d^	80.7^d^	0.82	P < 0.01
∆9-DI (16)	11.1^a^	11.7^a^	15.9^b^	18.6^c^	20.9^d^	21.2^d^	0.61	P < 0.01
Total DI	49.6^a^	49.7^a^	54.9^b^	58.2^c^	61.0^d^	60.8^d^	0.52	P < 0.01
Elongase index	0.62^d^	0.62^d^	0.57^c^	0.52^b^	0.47^a^	0.49^a^	0.019	P < 0.05
Thioesterase index	30.7^d^	29.6^d^	28.2^c^	26.6^b^	24.9^a^	25.3^a^	0.56	P < 0.05
∆5 + ∆6 desaturase index	16.7	17.1	16.4	16.5	17.1	17.1	0.53	P > 0.05

*T1 (no BFD/MOS/BMD), T2 (20 mg BMD/kg diet), T3 (0.1% MOS + 10^6^ cfu BFD/g feed), T4 (0.1% MOS + 10^7^ cfu BFD/g feed), T5 (0.2% MOS + 10^6^ cfu BFD/g feed), T6 (0.2% MOS + 10^7^ cfu BFD/g feed).

*BMD* Bacitracin methylene disalicylate, *DI* Desaturase index, *SEM* Standard error of mean.

Mean values bearing the same superscripts in a row do not differ significantly.

The health related indices measured in chicken breast and thigh meat were significantly influenced by BFD and MOS supplementation except ω-6: ω-3 PUFA ratio (Table 4). The PUFA:SFA ratio, MUFA:SFA ratio, UFA:SFA ratio (P < 0.01), DFA content, and h/H ratio (P < 0.05) exhibited a progressive increase; and S/P, AI, TI (P < 0.01), and HFA content (P < 0.05) revealed a progressive decrease from treatment T1 (control) to T6. But, no significant differences were observed between treatment group T1 and T2; and T5 and T6. Similar trend in lipid metabolism and health indices pattern was observed at 21 days of age (data not shown).

**Table 4:** Effect of dietary supplementation of *Bifidobacterium bifidum* (BFD) and Mannan-oligosaccharides (MOS) on health related indices of broiler chicken meat (n = 6 × 2).

Indices	T1*	T2*	T3*	T4*	T5*	T6*	SEM	P value
**Breast**								
ω-6: ω-3 PUFA ratio	6.71	6.52	6.46	6.33	5.96	6.02	0.603	P > 0.05
PUFA:SFA ratio	0.44^a^	0.45^a^	0.51^b^	0.57^c^	0.62^d^	0.62^d^	0.003	P < 0.01
MUFA:SFA ratio	0.82^a^	0.86^a^	1.00^b^	1.19^c^	1.42^d^	1.43^d^	0.039	P < 0.01
UFA:SFA ratio	1.27^a^	1.31^a^	1.50^b^	1.75^c^	2.04^d^	2.06^d^	0.041	P < 0.01
Saturation index (S/P)	0.76^d^	0.74^d^	0.64^c^	0.55^b^	0.47^a^	0.47^a^	0.023	P < 0.01
Atherogenic index (AI)	0.51^d^	0.50^d^	0.44^c^	0.38^b^	0.34^a^	0.33^a^	0.009	P < 0.01
Thrombogenic index (TI)	1.24^d^	1.19^d^	1.04^c^	0.89^b^	0.77^a^	0.76^a^	0.027	P < 0.01
DFA (%)	70.2^a^	70.6^a^	72.6^b^	74.5^c^	76.2^d^	76.4^d^	0.43	P < 0.05
HFA (%)	26.2^d^	25.9^d^	23.8^c^	21.9^b^	20.2^a^	19.8^a^	0.29	P < 0.05
h/H ratio	1.96^a^	2.01^a^	2.29^b^	2.61^c^	2.96^d^	3.01^d^	0.043	P < 0.05
**Thigh**								
ω-6: ω-3 PUFA ratio	4.16	4.13	4.25	4.24	4.20	4.16	0.121	P > 0.05
PUFA:SFA ratio	0.41^a^	0.43^a^	0.49^b^	0.53^c^	0.55^d^	0.55^d^	0.013	P < 0.05
MUFA:SFA ratio	0.97^a^	0.98^a^	1.19^b^	1.36^c^	1.51^d^	1.51^d^	0.053	P < 0.01
UFA:SFA ratio	1.38^a^	1.40^a^	1.68^b^	1.88^c^	2.07^d^	2.06^d^	0.029	P < 0.01
Saturation index (S/P)	0.70^d^	0.69^d^	0.58^c^	0.51^b^	0.47^a^	0.47^a^	0.012	P < 0.01
Atherogenic Index (AI)	0.48^c^	0.48^c^	0.41^b^	0.38^ab^	0.36^a^	0.36^a^	0.012	P < 0.05
Thrombogenic Index (TI)	1.08^d^	1.06^d^	0.90^c^	0.81^b^	0.74^a^	0.74^a^	0.019	P < 0.01
DFA (%)	71.7^a^	71.9^a^	73.9^b^	74.8^bc^	75.3^c^	75.6^c^	0.40	P < 0.05
HFA (%)	25.0^d^	24.8^d^	22.8^c^	21.8^b^	21.2^a^	21.0^a^	0.21	P < 0.05
h/H ratio	2.09^a^	2.12^a^	2.45^b^	2.64^c^	2.78^d^	2.80^d^	0.023	P < 0.05

*T1(no BFD/MOS/BMD), T2 (20 mg BMD/kg diet), T3 (0.1% MOS + 10^6^ cfu BFD/g feed), T4 (0.1% MOS + 10^7^ cfu BFD/g feed), T5 (0.2% MOS + 10^6^ cfu BFD/g feed), T6 (0.2% MOS + 10^7^ cfu BFD/g feed).

*BMD* Bacitracin methylene disalicylate, *PUFA* Poly-unsaturated fatty acid, *SFA* Saturated fatty acids, *MUFA* Mono-unsaturated fatty acids, UFA: Unsaturated fatty acids, *DFA* Desirable fatty acids, *HFA* Hypercholesterolaemic fatty acids, *h/H* hypocholesterolemic/hypercholesterolemic ratio, *SEM* Standard error of mean.

Mean values bearing the same superscripts in a row do not differ significantly.

### Serum lipid chemistry and health related indices

All the serum lipid parameters of broiler chicken measured in this study were significantly (P < 0.01) affected by BFD and MOS supplementation (Table 5). The serum TG and TC concentrations revealed a decreasing trend and serum HDL C an increasing trend from T1 (control) to T6. Similarly, the serum health indices—CRR, AC, and AIP exhibited a decreasing trend from T1 (control) to T6. However, for all the serum indices measure no significant differences were observed between treatment group T1 and T2; and T5 and T6.

**Table 5:** Effect of dietary supplementation of *Bifidobacterium bifidum* (BFD) and Mannan-oligosaccharides (MOS) on serum lipid chemistry and health related indices of broiler chicken (n = 6 × 2).

Indices	T1*	T2*	T3*	T4*	T5*	T6*	SEM	P value
TG (mg/dl)	127^d^	126^d^	123^c^	119^b^	114^a^	113^a^	1.16	P < 0.01
TC (mg/dl)	97.3^d^	98.6^d^	93.6^c^	87.2^b^	82.4^a^	82.5^a^	1.29	P < 0.01
HDL C (mg/dl)	50.9^a^	52.0^a^	55.9^b^	58.1^c^	61.7^d^	60.3^d^	0.69	P < 0.01
CRR	1.91^d^	1.89^d^	1.67^c^	1.50^b^	1.34^a^	1.37^a^	0.053	P < 0.01
AC	0.91^d^	0.89^d^	0.67^c^	0.50^b^	0.34^a^	0.37^a^	0.041	P < 0.01
AIP	0.40^d^	0.39^d^	0.34^c^	0.31^b^	0.27^a^	0.27^a^	0.013	P < 0.01

*T1 (no BFD/MOS/BMD), T2 (20 mg BMD/kg diet), T3 (0.1% MOS + 10^6^ cfu BFD/g feed), T4 (0.1% MOS + 10^7^ cfu BFD/g feed), T5 (0.2% MOS + 10^6^ cfu BFD/g feed), T6 (0.2% MOS + 10^7^ cfu BFD/g feed).

*BMD* Bacitracin methylene disalicylate, *TG* Triglyceride, *TC* Total cholesterol, *HDL C* High density lipoprotein cholesterol, *CRR* Cardiac risk ratio, *AC* Atherogenic coefficient, *AIP* Atherogenic index of plasma, *SEM* Standard error of mean

Mean values bearing the same superscripts in a row do not differ significantly.

## Discussion

In this study, a nutrigenomic approach was adopted to understand the molecular mechanisms behind the effects of dietary MOS and BFD supplementation on lipid metabolism in broiler chicken. Since modern day broiler chickens are hyperphagic and thus, more prone to obesity they are considered as models of choice to study lipid metabolism and their consequent effects in response to dietary synbiotic supplementation^3^. Liver was chosen for the transcriptome analysis because it is a major metabolic organ of the body involved in lipid metabolism^22^. Lipid deposition is the function of balance between the lipolysis and lipogenesis in the body^14^ and alteration in the expression pattern of genes involved in lipid metabolism in response to dietary synbiotics affect the lipid deposition in broiler chicken^3^. The key enzymes involved in lipogenesis are ACC, FAS, ME, and SCD-1 which are regulated by SERBP-1 and the key enzymes involved in lipolysis are PPAR-α and AMPKα-1^2,3,23^.

The present study revealed lower fat percentage of chicken meat from birds supplemented with 0.2% MOS along with either 10^6^ or 10^7^ CFU BFD/g feed. The possible determinants of lower fat percentage of chicken meat in the present study could be decline in the synthesis of lipids due to the down regulation of ACC, FAS, ME, and SERBP-1, lesser transport and deposition of synthesised lipids due to down regulation of apoB100, and enhanced oxidation or inhibition of lipogenesis due to upregulation of PPAR-α and AMPKα-1 expression in chicken liver. The process of lipogenesis is initiated by ACC by catalysing the rate-limiting step of carboxylation of acetyl-CoA to malonyl-CoA followed by a series of repetitive reaction by FAS^3,24^. During this process of lipogenesis FAS requires a continuous supply of reducing agent NADPH which is provided via oxidative decarboxylation of malic acid to pyruvic acid and CO_2_by ME^25,26^. In chicken, synbiotic supplementation down regulates the hepatic expression of SERBP-1c, a lipogenic nuclear transcriptional regulator that directly regulates the expressions of ACC, FAS, and ME^3,25^. On the other hand increased hepatic expression of PPAR-α was observed in broiler chicken due to dietary synbiotic supplementation^3,27^ which upregulates the expression of fatty acid catabolism genes, such as Carnitine palmitoyl transferase-1 and Acyl CoA oxidase-1, resulting in enhanced fatty acid β-oxidation in mitochondria^28^. Another mechanism put forward by earlier researchers as a cause of reduced body fat deposition is stimulation of farnesoid X receptor–PPAR-α–acyl CoA oxidase pathway in laying hens^14^ and mice^29^. Furthermore, the upregulation of AMPKα has been reported to decrease FAS expression by negatively regulating the expression of SERBP-1 in avian species^30^. Therefore, the consequent effect of SERBP-1c down regulation and upregulation of PPAR-α and AMPKα under dietary synbiotic supplementation is lower deposition of fat content in broiler chicken^2,3^ as observed in the present study. Another protein involved in lipid metabolism and investigated in this study was apoB100 protein. It is directly involved in transport and deposition of lipids in tissues^31^ and upregulation in its expression results in greater fat accumulation in broiler chicken^32^. Similar to the results of present study, synbiotic supplementation has been reported to down regulate the hepatic gene expression of apoB100 protein with the corresponding decline in muscle fat percentage^3^. Therefore, down regulation of hepatic apoB100 expression by synbiotic supplementation can be another possible mechanism of fat reduction in broiler chicken. However, it is noteworthy to state that lipid synthesis and its consequent deposition also depends on the age of birds and older chicken tend to deposit more fat in their body^33^. Corresponding to this age dependence of lipid deposition in chicken present study revealed the up regulation of PPAR-α and AMPKα-1 expression, and down regulation of SERBP-1 expression at 21 days of age but not at 42 days of age. It indicates that in young chicken rate of lipogenesis and lipid deposition is lower compared to adult birds^3,34^.

In the present study, ∆9-DI (18), ∆9-DI (16), and total DI increased significantly in birds supplemented with 0.2% MOS along with either 10^6^ or 10^7^ CFU BFD/g feed by mediating the up regulation of SCD-1 which catalyses the biosynthesis of MUFA from corresponding SFA^3,35^. Therefore, decrease in palmitic acid, stearic acid, and total SFA content; and increase in palmitoleic acid, oleic acid, and total MUFA content of chicken meat can be associated with up-regulation of SCD-1 expression. Lower thioesterase and elongase indices of chicken meat observed in the present study could be because of increased conversion of palmitic and stearic acids to their unsaturated counterparts under the influence of SCD-1 upregulation. The ∆5 + ∆6 desaturase indexof chicken meat, associated with catalysis of PUFAs synthesis, did not reveal any significant effect of synbiotic supplementation. This non-significant effect of synbiotic supplementation on ∆5 + ∆6 desaturase index and in turn on the PUFA content of chicken meat was also reported by Dev et al.^3^. On the similar pattern, the health indices of chicken meat detailed in this study were maximally improved in birds supplemented with 0.2% MOS along with either 10^6^ or 10^7^ CFU BFD/g feed because of increased MUFA content at the cost of SFA content of chicken meat which happened under the influence of SCD-1 up regulation. Other genes involved in lipid metabolism and studied in this experiment did not reveal any significant effect on health indices of chicken meat because of their non-significant effect on the fatty acid profile^3^.

Serum lipid metabolites such as triglycerides, total cholesterol, and other lipoproteins are sensitive indicators of lipid metabolism rate in chicken^3,36^. In the present study, serum TG, and TC decreased, whereas, serum HDL C increased in birds supplemented with 0.2% MOS along with either 10^6^ or 10^7^ CFU BFD/g feed. This hypocholesterolemic and hypolipidemic effect of synbiotic supplementation in broiler chicken resulted in improved serum health indices – CRR, AC, and AIP which was also reported by^3^ in broiler chicken in response to synbiotic supplementation. Improved serum lipid profile has been reported earlier in broiler chicken due to synbiotic supplementation^2,3,37,38^. Various mechanism of lipid lowering effects due to probiotics have been put forward, such as enhancement of deconjugation of bile acids^39^ which diverts more cholesterol towards synthesis of bile acids, intestinal conversion of cholesterol to coprostanol^40^, and subsequent excretion via faeces^3^. Also, the inhibition of 3-hydroxyl-3-methylglutaryl-coenzyme (HMG-CoA) reductase has been linked to cholesterol lowering effect of probiotics^9^. On the other hand, the lipid lowering effects of prebiotics have been ascribed to their ability to increase gut viscosity and mucus layer thickness which in turn inhibits cholesterol uptake from intestines; and further has been reported to enhance cholesterol breakdown^3,13^.

In conclusion, this study establishes that reduction in fat content of broiler chicken meat under the influence of synbiotic supplementation in chicken feed occurs by three possible mechanisms—reduction in rate of lipogenesis by down regulation of ACC, FAS, ME, and SERBP-1 expression, reduced transport and deposition of lipids in tissues by down regulation of apoB100 expression, and enhancement of β-oxidation of body lipids by upregulation of PPAR-α and AMPKα-1 expression in chicken liver. The ∆9-desaturation of chicken meat increased significantly under the influence of synbiotic supplementation by mediating the upregulation of SCD-1 expression which consequently resulted in increase of MUFA content at the cost of SFA content and in turn improved certain health indices of chicken meat. Further, the synbiotic supplementation in chicken feed produced hypocholesterolemic and hypolipidemic effect which improved the serum health indices of broiler chicken.

## Materials and methods

### Animal ethics compliance

This study was approved and carried out according to the guidelines of Institutional Animal Ethics Committee (IEAC) of Central Avian Research Institute, Izatnagar. The study was carried out in compliance with the Animal Research: Reporting of In Vivo Experiments (ARRIVE) guidelines.

### Experimental design

In this study a total of 288 one-day-old CARIBRO Vishal with considerable uniformity in body weight were obtained from the hatchery of Central Avian Research Institute, Izatnagar. The antibiotic bacitracin methylene disalicylate (BMD), prebiotic mannan-oligosaccharides (MOS), and probiotic *Bifidobacterium bifidum* (BFD) were added to corn-soybean meal basal diet of chicken to form six treatment groups—T1 (negative control diet), T2 (positive control, 20 mg BMD/kg diet), T3 (0.1% MOS + 10^6^ cfu BFD/g feed), T4 (0.1% MOS + 10^7^ cfu BFD/g feed), T5 (0.2% MOS + 10^6^ cfu BFD/g feed), and T6 (0.2% MOS + 10^7^ cfu BFD/g feed). The details of ingredients and nutrient composition of basal diet is shown in Supplementary 1. Each treatment group was allotted 48 birds in six replicates with eight birds in each. The diets in all the treatment groups were similar in energy, protein, and fatty acid profile to avoid potential confounding effects on the results of the study. BMD, with a certified 44% bacitracin activity, was purchased from ALPHARMA Animal Health Division New Jersey- USA and MOS was purchased from Kothari Fermentation & Biochem Ltd. India. BFD (UBBB-55), with strain number MTCC 5398, was purchased from Unique Biotech Ltd. India. It is of healthy human fecal origin in the form of cream to brown coloured powder with characteristic odour and certified stable at room temperature. The *E. coli* and Salmonella species per 10 g powder; and *Staphylococcus aureus* and *Pseudomonas aeruginosa* per g powder are certified to be absent with yeast and mould count not more than 100 cfu/g. To ensure the exact dose of probiotic, the concentration of live bacteria in BFD powder was verified by culture-based counting. Bifidobacterium Selective Count Agar Base (BSC Propionate Agar Base) was used for enumeration of BFD by making a serial dilution of BFD powder in sterile phosphate buffer saline. The dilution showing most visible and countable colony forming units on the agar was replicated six times and an average concentration was calculated at this dilution. The birds were fed ad libitum with free access to clean drinking water for an experimental period of 42 days.

For the analysis of lipid deposition and the consequent effects in broiler chicken in response to MOS and BFD supplementation sampling was done at 21 and 42 days of age. Six birds were sacrificed (one bird from each replicate with equal number of males and females) from each treatment group after 12 h of fasting and fresh breast and thigh meat samples (5 g) without skin (from same place) were collected in duplicate for fatty acid profile analysis. From the same birds liver samples (1 g) from similar areas were also collected and stored in RNAlater to analyze the expression profile of genes involved in lipid deposition in broiler chicken. Further, blood samples (2 ml) were collected from the same birds in duplicate in test tubes without anticoagulant. The serum was extracted from the blood samples and stored at − 20 °C till lipid profile analysis.

### Hepatic RNA extraction and expression of genes involved in lipid metabolism

From the liver samples RNA extraction was done by using Trizol reagent (INVITROGEN, Carlsbad, CA, USA) following the instructions of manufacturer. Nanodrop (NANO DROP 1000, thermo-scientific, Singapore) was used to check RNA sample concentration at 260 nm, ethidium bromide staining was used to check RNA integrity by agarose gel-electrophoresis, and RNA sample purity was checked by UV spectrophotometry (OD_260_/OD_280_). The reverse transcription of extracted RNA samples (5 µg) was carried out along with a negative control by using ‘Revert Aid First strand cDNA synthesis kit’ (MBI Fermentas, Hanover, MD, USA) for cDNA synthesis. Again, the concentration of synthesized cDNA samples was determined by using Nanodrop at 260 nm (NANO DROP 1000, thermo-scientific, Singapore) and thereafter, stored frozen at − 20 °C till further use.

The expression analysis of major enzymes involved in lipid metabolism such as acetyl carboxylase (ACC), fatty acid synthase (FAS), malic enzyme (ME), apolipoprotein B100 (apoB100), sterolregulatory element binding protein-1 (SREBP-1), Stearoyl-CoA (Δ9) desaturase-1 (SCD-1), Peroxisome proliferator activated receptor- α (PPAR-α), and AMP-activated protein kinase α-1 (AMPKα-1) was carried out by using their specific primers against β-actin as housekeeping gene. Oligonucleotide primer pairs used for lipid metabolism related gene expression study shown in Table 6. The primers were synthesized commercially by Integrated DNA technologies (New Delhi). The ideal conditions for each gene specific primer pair was worked out through gradient PCR in Gradient thermal cycler (BIO-RAD, USA). Real time qPCR analysis was performed by IQ5 Cycler system (BIO-RAD, Hercules, CA, USA) with SYBER Premix Ex Taq reagent Kit (TAKARA Biotech, Japan). The qRT-PCR conditions were as follows: initial denaturation at 95 °C for 15 min; followed by 40 cycles of subsequent denaturation at 95 °C for 30 s, annealing at 58 °C for 30 s, and extension at 72 °C for 30 s. All the samples were run in triplicate in nuclease-free 8 tube-strips with optically clear flat caps (AXYGEN Scientific, Inc. USA). The gene expression levels were normalized to *β-actin* and the results were analyzed by 2^–ΔΔCT^ method^41^.

**Table 6:** Nucleotide sequences of specific PCR primers of different genes.

Gene	Primer sequence (5′ → 3′)	Product size (bp)	Annealing temperature (°C)	Gene bank accession number
β-Actin	F-TGCGTGACATCAAGGAGAAG R-TGCCAGGGTACATTGTGGTA	300	58.2	L08165
FAS	F-CTATCGACACAGCCTGCTCCT R-CAGAATGTTGACCCCTCCTACC	107	55.9	J03860
ACC	F-AATGGCAGCTTTGGAGGTGT R-TCTGTTTGGGTGGGAGGTG	136	55.2	NM_205505
ME	F-TGCCAGCATTACGGTTTAGC R-CCATTCCATAACAGCCAAGGTC	175	53.9	NM204303
SCD-1	F-TCCCTTCTGCAAAGATCCAG R-AGCACAGCAACACCACTGAG	402	56.5	X60465
apoB100	F-GACAGAAGGTTATGGAGC R-TCTGAGTGCCTGTCTGCT	365	54	NM_001044633.1
SREBP-1	F-GCAGAAGAGCAAGTCCCTCAA R-TCGGCATCTCCATCACCTC	104	55.1	AY029224
PPARα	F-TGGACGAATGCCAAGGTC R-GATTTCCTGCAGTAAAGGGTG	813	58.9	AF163809
AMPKα-1	F-CGGAGATAAACAGAAGCACGAG R-CGATTCAGGATCTTCACTGCAAC	266	58.3	DQ302133

*ACC* Acetyl-CoA carboxylase, *FAS* Fatty acid synthase, *ME* Malic enzyme, *SREBP* Sterol regulatory element binding protein, *SCD-1* Stearoyl-CoA (Δ9) desaturase, *PPAR-α* Peroxisome proliferator activated receptor, *AMPK* AMP-activated protein kinase, *apoB100* Apolipoprotein B100.

### Chromatography and fatty acid profile

Fatty acid methyl esters (FAMEs) were prepared directly from meat samples as described by O’Fallon et al.^42^ and C13:0 ME was used as internal standard. The fatty acid composition of FAMEs was analysed by capillary gas chromatograph by following the standardised laboratory protocol^43^. Further, fat percentage of meat samples was estimated with the help of Soxhlet extraction apparatus by refluxing of meat samples (2 g) in petroleum ether for 5–6 h^44^.

### Lipid metabolism indices

The lipid metabolism indices were calculated from the data of fatty acid profile of meat samples. The measurement of enzyme activity indices determine the extent of desaturation activities in tissues and the conversion of fatty acids to relatively longer chain fatty acids. The activity of steroyl-CoA desaturases convert saturated fatty acids (SFA) to monounsaturated fatty acids (MUFA) which were calculated by relating the percentage of product to the correspondingsubstrate^45^:

$$\Delta^{{9}} - {\text{desaturase }}\left( {{18}} \right){\text{ index }}\left\{ {\Delta^{{9}} - {\text{DI }}\left( {{18}} \right)} \right\} \, = { 1}00 \, \left[ {{\text{C18}}{:}{1}/\left( {{\text{C18}}{{:}}{1 } + {\text{ C18}}{:}0} \right)} \right]$$

$$\Delta^{{9}} - {\text{desaturase }}\left( {{16}} \right){\text{ index }}\left\{ {\Delta^{{9}} - {\text{DI }}\left( {{16}} \right)} \right\} \, = { 1}00 \, \left[ {{\text{C16}}{:}{1}/\left( {{\text{C16}}{:}{1 } + {\text{ C16}}{:}0} \right)} \right]$$

$${\text{Total }}\Delta^{{9}} - {\text{DI }} = { 1}00 \, \left[ {\left( {{\text{C16}}{:}{1 } + {\text{ C18}}{:}{1}} \right)/\left( {{\text{C16}}{:}{1 } + {\text{ C16}}{:}0 \, + {\text{ C18}}{:}{1 } + {\text{ C18}}{:}0} \right)} \right]$$

The conversion of essential fatty acids (EFA)—linoleic acid (LA) and ALA was measure in terms of Δ^5^+ Δ^6^ desaturase index and the conversion of myristic acid (C14:0) to palmitic acid (C16:0) and further to steric acid (C18:0) was measured by thioesterase and elongase indices, respectively^46^:

$$\Delta^{{5}} + \, \Delta^{{6}} - {\text{desaturase index }} = { 1}00 \, [\{ {\text{C2}}0{:}{\text{2}}\omega - {6 } + {\text{ C2}}0{:}{\text{4}}\omega - {6 } + {\text{ EPA }} + {\text{ C22}}{:}{\text{5}}\omega - {3 } + {\text{ DHA}}\} \, \div \, \{ {\text{C18}}{:}{\text{2}}\omega - {6 } \; \left( {{\text{LA}}} \right) \, + {\text{ ALA }} + {\text{ C2}}0{:}{\text{2}}\omega - {6 } + {\text{ C2}}0{:}{\text{4}}\omega - {6 } + {\text{ EPA }} + {\text{ C22}}{:}{\text{5}}\omega - {3 } + {\text{ DHA}}\} ]$$

$${\text{Elongase index }}\left( {{\text{EI}}} \right) \, = {\text{ C18}}{:}0/{\text{C16}}{:}0$$

$${\text{Thioesterase index }}\left( {{\text{TI}}} \right) \, = {\text{ C16}}{:}0/{\text{C14}}{:}0$$

### Health indices of meat

Various health indices, useful for evaluating the nutritional value and healthiness of the chicken meat, were also calculated from the data of fatty acid profile. The saturated fatty acids (SFA) are considered as proatherogenic and prothrombogenic fatty acids, whereas, unsaturated fatty acids (UFA) are considered as antiatherogenic and antithrombogenic fatty acids. The common health indicators of chicken meat ω-6 to ω-3 PUFA, PUFA to SFA, MUFA to SFA, and UFA to SFA ratios were calculated. The other indicators of health value of chicken meat calculated in this study were saturation index (S/P), atherogenic index (AI), thrombogenic index (TI)^46^, desirable fatty acid (DFA) content, hypercholesterolemic fatty acids (HFA), and hypocholesterolemic to hypercholesterolemic fatty acid ratio (h/H)^47^.

$$\begin{aligned} & {\text{S}}/{\text{P }} = \, \left( {{\text{C14}}{:}0 \, + {\text{ C16}}{:}0 \, + {\text{ C18}}{:}0} \right)/ \left( {{\text{MUFA }} + {\text{ PUFA}}} \right) \\ & {\text{AI }} = \, \left( {{\text{C12}}{:}0 \, + { 4 } \times {\text{ C14}}{:}0 \, + {\text{ C16}}{:}0} \right)/\left( {{\text{MUFA }} + {\text{ PUFA}}} \right) \\ & {\text{TI }} = \, \left( {{\text{C14}}{:}0 \, + {\text{ C16}}{:}0 \, + {\text{ C18}}{:}0} \right)/\left[ {\left( {{\text{MUFA }} + \omega - {\text{6 PUFA}}} \right)/{2 } + { 3 } \times \omega - {\text{3 PUFA }} + \omega - {3}{:} \omega - {6}} \right] \\ & {\text{DFA }} = {\text{ UFA }} + {\text{ C18}}{:}0 \\ & {\text{HFA }} = \, \left( {{\text{sum of C12}}{:}0,{\text{ C14}}{:}0,{\text{ and C16}}{:}0} \right) \\ & {\text{h}}/{\text{H }} = \, \left( {{\text{C18}}{:}{1 } + {\text{ PUFA}}} \right)/\left( {{\text{C14}}{:}0 \, + {\text{ C16}}{:}0} \right) \\ \end{aligned}$$

### Serum lipid chemistry and health related indices

The serum samples collected were used for the estimation of serum triglyceride (TG), total cholesterol (TC), and HDL cholesterol by using Span Diagnostic kits as per manufacturer’s instructions. Further, the atherogenic indices of serum Cardiac Risk Ratio (CRR), Atherogenic Coefficient (AC), and Atherogenic Index of Plasma (AIP) were calculated based on the serum lipid profile^48^.

$$\begin{aligned} & {\text{CRR }} = {\text{ Total cholesterol}}/{\text{HDL cholesterol}} \\ & {\text{AC }} = \, ({\text{Total cholesterol}}{-}{\text{ HDL cholesterol}})/{\text{ HDL cholesterol}} \\ & {\text{AIP }} = {\text{ Log }}\left( {{\text{Triglycerides}}{/}{\text{HDL cholesterol}}} \right) \\ \end{aligned}$$

### Statistical analysis

Data are presented as mean ± SEM and each sampled bird was taken as an experimental unit. Following the General Linear Model procedure, one-way ANOVA (SPSS software-20) was conducted to determine the dietary effects on the parameters measured above. The significant mean differences were separated by Tukey post-hoc analysis and significance level was set at P < 0.05.

### Ethical approval

All applicable institutional guidelines for the care and use of animals were followed. The experimental procedures carried out in this study were approved by the Institutional Animal Ethics Committee (IEAC) following the guidelines of ‘Committee for the Purpose of Control and Supervision of Experiments on Animals (CPCSEA) 2012’established under the “Prevention of Cruelty of Animals Act 1960” of Indian Penal Code (18 September 2017/Project No. 11). The study was carried out in compliance with the Animal Research: Reporting of In Vivo Experiments (ARRIVE) guidelines.

## Supplementary Information

Supplementary Information.

## Data Availability

The datasets analysed during the current study are available from the corresponding author on reasonable request.
