# Supplementary material for: Hepatic transcriptome analysis reveals altered lipid metabolism and consequent health indices in chicken supplemented with dietary Bifidobacterium bifidum and mannan-oligosaccharides
Source: Sci Rep. 2021 Sep 9;11:17895. doi: 10.1038/s41598-021-97467-1 (PMC8429770; doi:10.1038/s41598-021-97467-1)
Supplement: Supplementary file 1 — Supplementary Information. [file 41598_2021_97467_MOESM1_ESM.doc]

**Supplementary table**

| Table S1: Ingredients and nutrient composition of broiler chicken diets | | | |
| --- | --- | --- | --- |
| Ingredients (g/kg) | Pre-starter  (0-7 days) | Starter  (8-21 days) | Finisher  (22-42 days) |
| Maize | 443 | 460 | 505 |
| Soybean (crude protein 44.6%) | 410 | 380 | 342 |
| Rapeseed meal | 30 | 30 | 30 |
| Fish meal | 50 | 50 | 30 |
| Vegetable oil | 42 | 55 | 65 |
| Limestone | 6.0 | 6.0 | 7.0 |
| Di-calcium Phosphate | 13.5 | 13.6 | 15.5 |
| Salt | 3.0 | 3.0 | 3.0 |
| DL-Methionine | 0.2 | 0.2 | 0.2 |
| TM premix^1^ | 1.0 | 1.0 | 1.0 |
| Vitamin premix^2^ | 1.5 | 1.5 | 1.5 |
| Vitamin B complex^3^ | 0.15 | 0.15 | 0.15 |
| Choline chloride | 0.50 | 0.50 | 0.50 |
| Nutrient composition of diets (Analysed) | | | |
| Crude protein (g/kg) | 231 | 220 | 200 |
| M Energy (Kcal/kg) | 3001 | 3101 | 3200 |
| Calcium (g/kg) | 10.0 | 10.0 | 10.0 |
| Available phosphorus (g/kg) | 4.9 | 4.8 | 4.6 |
| Lysine (g/kg) | 13.3 | 12.1 | 10.6 |
| Methionine (g/kg) | 5.0 | 5.0 | 4.6 |
| Fatty acid profile of diets |  |  |  |
| C14:0 | 1.60 | 1.69 | 1.81 |
| C16:0 | 32.4 | 32.9 | 33.5 |
| C16:1 | 9.8 | 9.6 | 10.1 |
| C18:0 | 0.75 | 1.10 | 0.83 |
| C18:1 ω-9 | 37.1 | 36.4 | 37.4 |
| C18:2 ω-6 | 14.9 | 15.0 | 13.2 |
| C18:3 ω-3 | 3.45 | 3.31 | 3.16 |
| SFA | 34.8 | 35.7 | 36.1 |
| MUFA^2^ | 46.9 | 46 | 47.5 |
| PUFA^3^ | 18.4 | 18.3 | 16.4 |
| ^1^Trace mineral mixture (100 g): FeSO_4_.7H_2_O 8 g, ZnSO_4_.7H_2_O 10 g, MnSO_4_.H_2_O 10 g, CUSO_4_.5H_2_O 1 g, KI 30 g  ^2^Vitamin premix (1 g): Vitamin A 82.5 IU, Vitamin E 50% 160 mg, Vitamin D3 12000 unit, Vitamin K 10 mg  ^3^Vitamin B complex (1 g): Vitamin B1 8 mg, Vitamin B2 50 mg, Vitamin B6 16 mg, Vitamin B12 80 mcg, Niacin 120 mg, Calcium panthotheonate 80 mg , L-lysine 10 mg, and DL- Methionine 10 mg | | | |
